# Role of postoperative radiotherapy in pT3N0 rectal cancer: A risk‐stratification system based on population analyses

**DOI:** 10.1002/cam4.1991

**Published:** 2019-02-04

**Authors:** Yun‐xia Huang, Yan‐zong Lin, Jin‐luan Li, Xue‐qing Zhang, Li‐rui Tang, Qing‐yang Zhuang, Fei‐fei Lin, Xi‐jin Lin, Jun‐xin Wu

**Affiliations:** ^1^ Department of Radiation Oncology Fujian Medical University Cancer Hospital Fujian Cancer Hospital Fuzhou China; ^2^ Department of Gastrointestinal Surgical Oncology Fujian Medical University Cancer Hospital Fujian Cancer Hospital Fuzhou China

**Keywords:** adjuvant radiotherapy, prognosis, pT3N0 rectal cancer, SEER

## Abstract

The impact of adjuvant radiotherapy in pT3N0 rectal cancer is controversial. We aimed to determine the risk factors for cancer‐specific survival (CSS) among these patients and to develop a risk‐stratification system to identify which of these patients would benefit from adjuvant radiotherapy. In this review of the Surveillance, Epidemiology, and End Results database (2010‐2014), we analyzed the data of pT3N0 rectal cancer patients who had not undergone neoadjuvant radiotherapy. Prognostic factors were identified using the Cox proportional hazards model, and risk scores were derived according to the *β* regression coefficient. A total of 1021 patients were identified from the database search. The overall 5‐year CSS was 86.31%. Multivariate analysis showed that age (*P* < 0.001), tumor differentiation (*P* = 0.044), number of nodes resected (*P* = 0.032), marital status (*P* = 0.005), and radiotherapy (*P* = 0.006) were independent prognostic factors for CSS. A risk‐stratification system composed of age, tumor differentiation, and number of nodes resected was generated. Low‐risk patients had better CSS than high‐risk patients (92.13% vs 72.55%, *P* < 0.001). The addition of radiotherapy to surgery doubled the CSS among the high‐risk patients (42.06% vs 91.26%, *P* = 0.001) but produced no survival benefit among the low‐risk patients (93.36% vs 96.38%, *P* = 0.182). Our risk‐stratification model based on age, tumor differentiation, and number of nodes resected predicted the outcomes of pT3N0 rectal cancer patients. This model could help identify patients who may benefit from adjuvant radiotherapy.

## 1 INTRODUCTION

Colorectal cancer is the fourth most common form of cancer and the second leading cause of cancer‐related deaths worldwide.^1^ Approximately, 35% of all colorectal cancers involve the rectum.^2^ Neoadjuvant chemoradiotherapy followed by total mesorectal excision is the standard treatment for locally advanced rectal cancer, as it offers the advantages of sphincter preservation and local control.^3^ However, due to the inaccuracy of preoperative staging, some patients, especially those with pT3N0 disease, additionally require adjuvant radiotherapy after undergoing surgery.^4^ These patients are characterized as having an intermediate risk of local recurrence.^5^

The role of adjuvant radiotherapy in patients with pT3N0 rectal cancer is controversial. Some studies have reported that adjuvant radiotherapy can be omitted for a subset of pT3N0 patients with favorable prognostic factors.^6^, ^7^ Another study has found that while the oncological outcomes of total mesorectal excision strongly depend on the surgeon's experience, the technique used, and the location of the cancer,^8^ patients with pT3N0 disease still might benefit from perioperative radiotherapy.^9^ It is highly desirable to offer adjuvant radiotherapy to only those patients who are most likely to benefit from it and to consider omitting radiotherapy in patients with a low risk of cancer‐related death. Thus, a scoring system that stratifies the risk of cancer‐related death in patients with pT3N0 rectal cancer would be useful to determine the necessity of adjuvant radiotherapy in this patient population.

Therefore, in this study, we aimed to identify the prognostic factors that determine cancer‐specific survival (CSS) in patients with pT3N0 rectal cancer and to develop a risk‐stratification model that could be used to identify which of these patients might benefit from adjuvant radiotherapy after surgical resection.

## 2 MATERIALS AND METHODS

### 2.1 Study design and ethics statement

This study was a review of data obtained from the Surveillance, Epidemiology, and End Results (SEER) database. The SEER program provides information on cancer statistics, including incidence, prevalence, and survival, covering approximately 28% of the American population.^10^ As SEER data are publicly available and involve anonymized patient medical records, patient consent and institutional review approval were not required for this review.

### 2.2 Search strategy and data extraction

**Figure 1:**
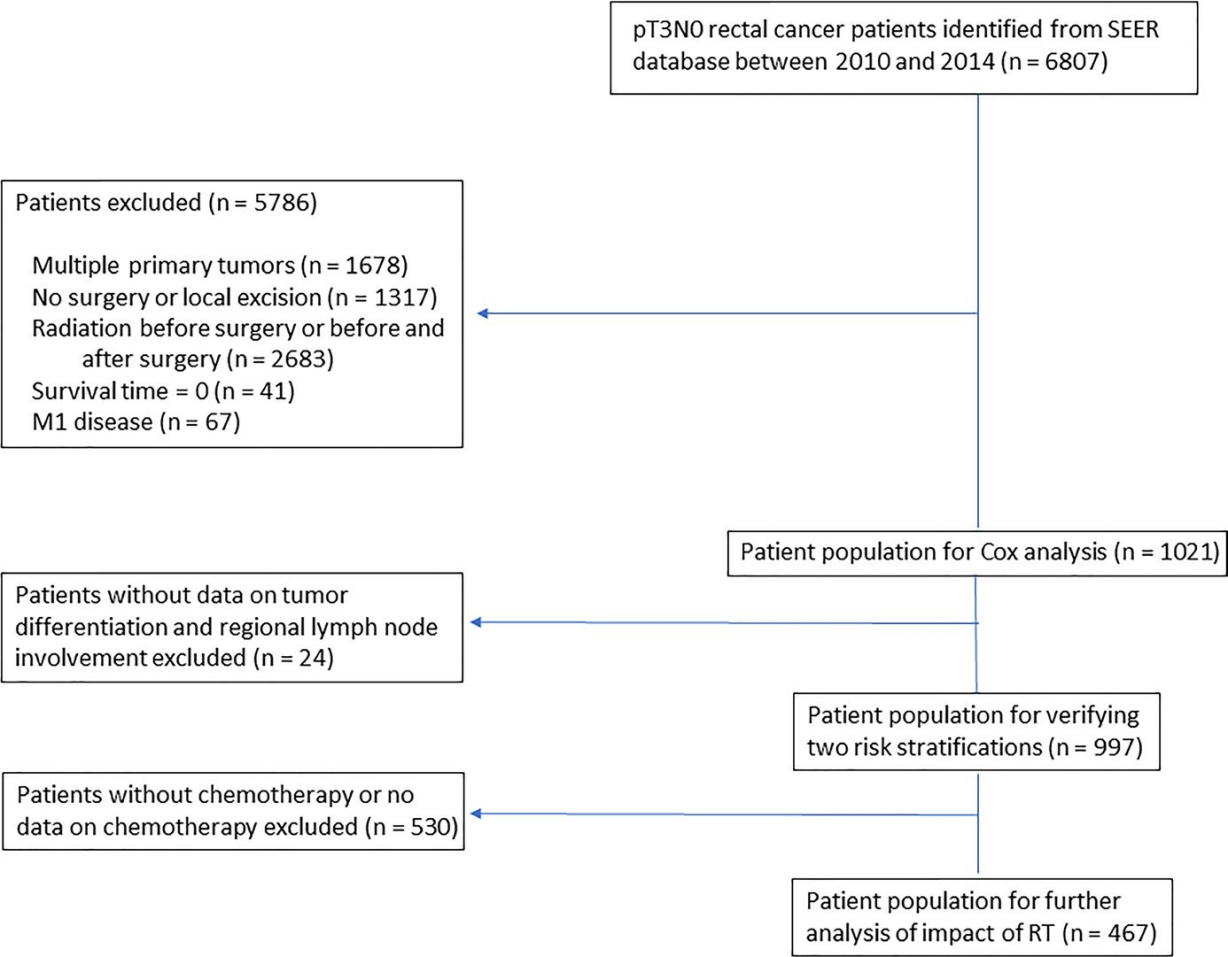
Flow chart of the search protocol and study design

We used the SEER‐Stat software (SEER*Stat 8.3.5) to retrieve the data of all patients who were diagnosed with rectal cancer (C20.9 Rectum, NOS) between 2010 and 2014. A total of 6807 patients had pT3N0 disease according to the 7th American Joint Committee on Cancer (AJCC) TNM staging system (Figure 1). Patients with multiple primary cancers (n = 1678), surgery type indicated as “local excision” or “no surgery performed” (n = 1317), or radiation sequence with surgery recorded as “radiation prior to surgery” or “radiation before and after surgery” (n = 2683) were excluded. We also excluded patients with survival time listed as “0” (n = 41) and patients with metastases (n = 67). Finally, data from 1021 patients, including 635 patients who underwent surgery alone and 386 patients who underwent surgery combined with adjuvant radiotherapy, were included in this study.

### 2.3 Statistical analysis

The primary endpoint of interest was CSS. The demographic and tumor characteristics of the patients who underwent surgery alone and those who underwent surgery plus radiotherapy were compared using the chi‐square (*χ* ^2^) test. Differences in survival rates were compared using the Kaplan‐Meier method. The Cox proportional hazards regression model was applied for multivariate analysis, and risk scores were derived using the *β* regression coefficient. The cutoff score impacting CSS was determined using the Cutoff Finder application (Jan Budczies, Germany).^11^ All statistical tests were performed using SPSS version 22.0 (IBM Corporation, Armonk, NY, USA). *P*‐values <0.05 were considered statistically significant.

## 3 RESULTS

### 3.1 Patient characteristics

We retrospectively analyzed the data of 1021 patients with pT3N0 rectal cancer from the SEER database. All patients had undergone radical surgery. The patient demographics and clinicopathological characteristics are summarized in Table 1. The median follow‐up time was 55.8 months (25.3‐84.2 months). Radiotherapy was more common among patients aged <70 years than among patients aged ≥70 years (*P* < 0.001), among patients with <12 nodes resected than among those with ≥12 nodes resected (*P* = 0.029), among patients who were married than among those who were single or widowed/divorced (*P* = 0.006), and among patients who underwent chemotherapy than among those who did not (*P* < 0.001). The CSS of the overall cohort was 86.31% (95% confidence interval [CI]: 81.23%‐90.10%). The CSS was significantly lower in the surgery‐alone group (79.67%, 95% CI: 70.27%‐86.39%) than in the surgery plus radiotherapy group (94.40%, 95% CI: 89.86%‐96.94%; *P* = 0.001).

**Table 1:** Demographic and tumor characteristics of 1021 rectal cancer patients

Variable	Total, *n* (%)	S, *n* (%)	S + RT, *n* (%)	*P*
	1021 (100)	635 (62.19)	386 (37.81)	
Year of diagnosis				0.095
2010	210 (20.57)	125 (19.69)	85 (22.02)	
2011	219 (21.45)	126 (19.84)	93 (24.09)	
2012	211 (20.67)	130 (20.47)	81 (20.98)	
2013	193 (18.90)	122 (19.21)	71 (18.39)	
2014	188 (18.41)	132 (20.79)	56 (14.51)	
Race				0.078
White	831 (81.39)	528 (83.15)	303 (78.50)	
Black	75 (7.35)	38 (5.98)	37 (9.59)	
Other	115 (11.26)	69 (10.87)	46 (11.92)	
Marital status				0.006
Married	566 (55.44)	337 (53.07)	229 (59.33)	
Single/unmarried	155 (15.18)	88 (13.86)	67 (17.36)	
Divorced/widowed	250 (24.49)	178 (28.03)	72 (18.65)	
Unknown	50 (4.90)	32 (5.04)	18 (4.66)	
Insurance				0.023
Yes	971 (95.10)	611 (96.22)	360 (93.26)	
No	33 (3.23)	13 (2.05)	20 (5.18)	
Unknown	17 (1.67)	11 (1.73)	6 (1.55)	
Age				<0.001
<70 years	669 (65.52)	359 (56.54)	310 (80.31)	
≥70 years	352 (34.48)	276 (43.46)	76 (19.69)	
Gender				0.127
Male	588 (57.59)	354 (55.75)	234 (60.62)	
Female	433 (42.41)	281 (44.25)	152 (39.38)	
Tumor differentiation				0.142
Grade I/II	905 (88.64)	572 (90.08)	333 (86.27)	
Grade III/IV	99 (9.70)	55 (8.66)	44 (11.40)	
Unknown	17 (1.67)	8 (1.26)	9 (2.33)	
Histology				0.575
Adenocarcinoma	962 (94.22)	602 (94.80)	360 (93.26)	
Mucinous & SRCC	55 (5.39)	31 (4.88)	24 (6.22)	
Others	4 (0.39)	2 (0.31)	2 (0.52)	
Tumor size				0.07
≤5 cm	547 (53.57)	331 (52.13)	216 (55.96)	
>5 cm	420 (41.14)	276 (43.46)	144 (37.31)	
Unknown	54 (5.29)	28 (4.41)	26 (6.74)	
Number of nodes resected				0.029
<12	230 (22.53)	126 (19.84)	104 (26.94)	
≥12	784 (76.79)	505 (79.53)	279 (72.28)	
Unknown	7 (0.69)	4 (0.63)	3 (0.78)	
Carcinoembryonic antigen				0.08
≤5 ng/mL	365 (35.75)	229 (36.06)	136 (35.23)	
>5 ng/mL	243 (23.80)	137 (21.57)	106 (27.46)	
Unknown	413 (40.45)	269 (42.36)	144 (37.31)	
Circumferential radial margin				0.413
Negative	712 (69.74)	449 (70.71)	263 (68.13)	
Positive	140 (13.71)	80 (12.60)	60 (15.54)	
Unknown	169 (16.55)	106 (16.69)	63 (16.32)	
Perineural invasion				0.776
Negative	881 (86.29)	550 (86.61)	331 (85.75)	
Positive	72 (7.05)	42 (6.61)	30 (7.77)	
Unknown	68 (6.66)	43 (6.77)	25 (6.48)	
Chemotherapy				<0.001
Yes	540 (52.89)	508 (80.00)	32 (8.29)	
No	481 (47.11)	127 (20.00)	354 (91.71)	

S, surgery alone; S + RT, surgery combined with adjuvant radiotherapy; SRCC, signet ring cell carcinoma.

### 3.2 Subgroup analyses

**Figure 2:**
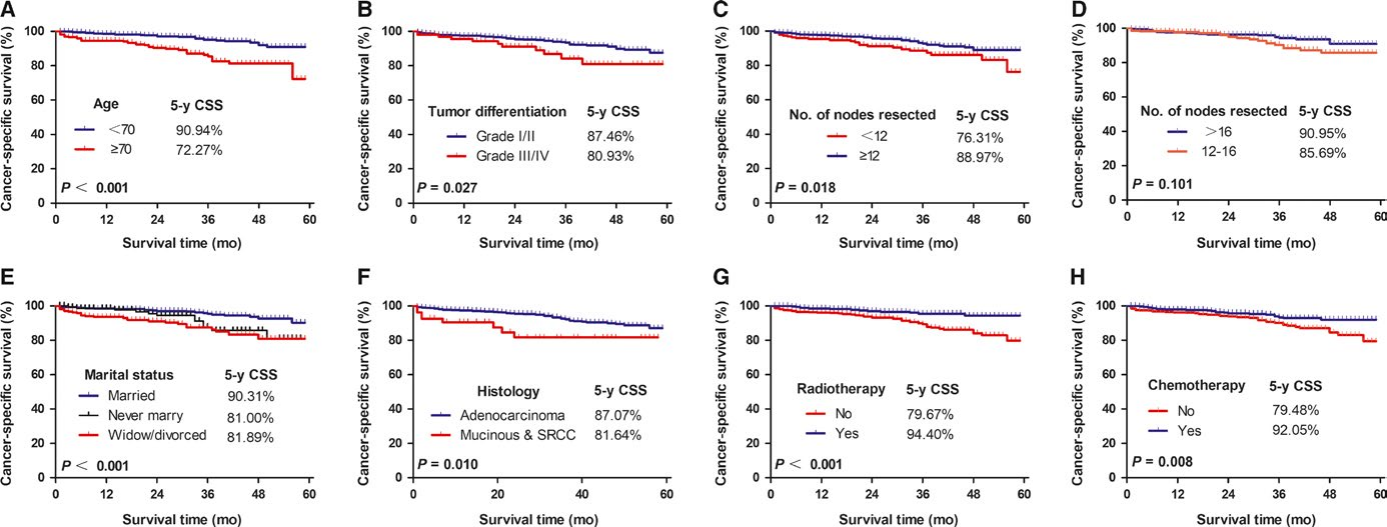
Kaplan‐Meier analysis of cancer‐specific survival according to (A) age (<70 vs ≥70 years, *P* < 0.001); (B) tumor differentiation (grade I/II vs grade III/IV, *P* = 0.027); (C) number of nodes resected (<12 vs ≥12, *P* = 0.018); (D) number of nodes resected (12‐16 vs >16, *P* = 0.101); (E) marital status (married vs never married vs widowed/divorced, *P* < 0.001); (F) histology (adenocarcinoma vs mucinous and signet ring cell carcinoma, *P* = 0.010); (G) radiotherapy (Yes vs No, *P* < 0.001); and (H) chemotherapy (Yes vs No, *P* = 0.008)

Kaplan‐Meier analysis showed that patients aged <70 years had a more favorable prognosis than those aged ≥70 years (CSS: 90.94% vs 72.27%, *P* < 0.001; Figure 2A). The CSS was 87.46% among patients with grade I or II cancer, and 80.93% among those with grade III or IV cancer (*P* = 0.027, Figure 2B). A better prognosis was observed in patients for whom ≥12 nodes had been resected than in patients for whom <12 nodes had been resected (CSS: 88.97% vs 76.31%, *P* = 0.018; Figure 2C). However, no survival difference was observed between the patients with 12‐16 nodes resected and those with >16 nodes resected (CSS: 85.69% vs 90.95%, *P* = 0.101; Figure 2D). Married patients had better outcomes than patients who were unmarried or widowed/divorced (*P* < 0.001; Figure 2E). Histological subtype was also associated with CSS, and patients with adenocarcinoma had a significantly better prognosis than did patients with mucinous or signet ring cell carcinoma (CSS: 87.07% vs 81.64%, *P* = 0.010; Figure 2F). The addition of radiotherapy following surgery was associated with a statistically significant improvement in CSS as compared to surgery alone (CSS: 79.67% vs 94.40%, *P* < 0.001; Figure 2G). Similarly, the addition of chemotherapy to surgery resulted in a better survival than surgery without chemotherapy (79.48% vs 92.05%, *P* = 0.008; Figure 2H).

### 3.3 Prognostic factors affecting CSS

Univariate analyses showed that age (*P* < 0.001), tumor differentiation (*P* = 0.031), number of nodes resected (*P* = 0.020), marital status (*P* < 0.001), histological subtype (*P* = 0.013), radiotherapy (*P* < 0.001), and chemotherapy (*P* = 0.009) were associated with CSS (Table 2).

**Table 2:** Univariate and multivariate analyses for 1021 pT3N0 rectal cancer patients

Variable	Univariate analysis		Multivariate analysis	
	HR (95% CI)	*P*	HR (95% CI)	*P*
Race				
White	Reference	0.391		
Black	1.401 (0.637‐3.079)	0.401		
Other	0.628 (0.251‐1.570)	0.320		
Marital status				
Married	Reference	<0.001	Reference	0.005
Single/unmarried	2.185 (1.081‐4.416)	0.030	2.857 (1.343‐6.079)	0.006
Divorced/widowed	3.176 (1.807‐5.582)	<0.001	2.357 (1.263‐4.400)	0.007
Insurance				
Yes/No	1.588 (0.498‐5.508)	0.434		
Age				
<70 vs ≥70 years	3.246 (1.984‐5.312)	<0.001	3.425 (1.844‐6.359)	<0.001
Gender				
Male/female	0.766 (0.464‐1.265)	0.298		
Tumor differentiation				
Grade I/II vs Grade III/IV	2.050 (1.068‐3.934)	0.031	2.078 (1.019‐4.240)	0.044
Histology				
Adenocarcinoma/Mucinous & SRCC	2.547 (1.215‐5.340)	0.013	1.530 (0.539‐4.339)	0.424
Tumor size				
≤5 vs >5 cm	0.863 (0.515‐1.446)	0.576		
Number of nodes resected				
<12 vs ≥12	0.549 (0.331‐0.911)	0.020	0.544 (0.312‐0.949)	0.032
Carcinoembryonic antigen				
≤5 vs >5 ng/mL	1.920 (0.987‐3.736)	0.055		
Circumferential radial margin				
Negative vs positive	1.220 (0.626‐2.378)	0.558		
Perineural invasion				
Negative vs positive	1.406 (0.605‐3.267)	0.428		
Radiotherapy				
Yes vs No	0.334 (0.182‐0.612)	<0.001	0.300 (0.128‐0.702)	0.006
Chemotherapy				
Yes vs No	0.509 (0.307‐0.845)	0.009	1.725 (0.819‐3.632)	0.151

SRCC, signet ring cell carcinoma.

Unknown data points were removed before performing statistical tests.

The risk factors identified by univariate analyses were included in multivariate analyses (Table 2). The following factors were found to be independently associated with a significantly increased risk of cancer death: age ≥70 years (hazard ratio [HR]: 3.425, 95% CI: 1.844‐6.359, *P* < 0.001), grade III or IV tumor (HR: 2.078, 95% CI: 1.019‐4.240, *P* = 0.044), <12 nodes resected (HR: 1.838, 95% CI: 1.054‐3.205, *P* = 0.032), and absence of radiotherapy (HR: 3.33, 95% CI: 1.425‐7.813, *P* = 0.006).

### 3.4 Establishment of risk‐stratification model

**Figure 3:**
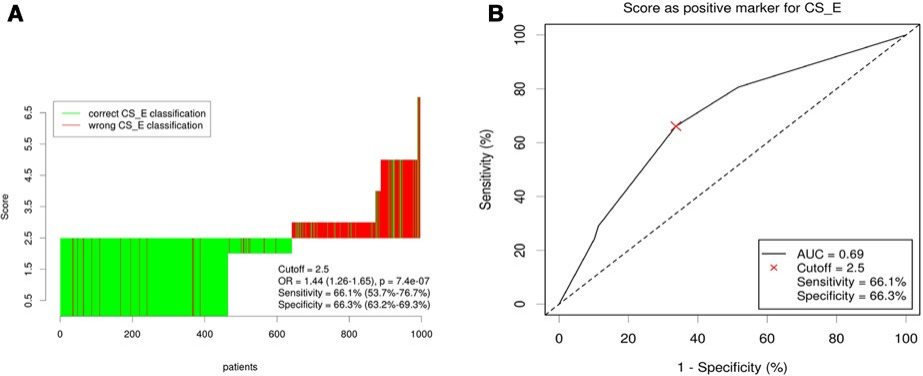
Distribution‐based cutoff optimization for risk score. (A) Waterfall plot of optimal dichotomization. Classification using the risk scores; the optimal cutoff was assessed for the event of cancer‐specific death. (B) Receiver operating characteristic curve of risk scores; the optimal cutoff was assessed for the event of death

**Figure 4:**
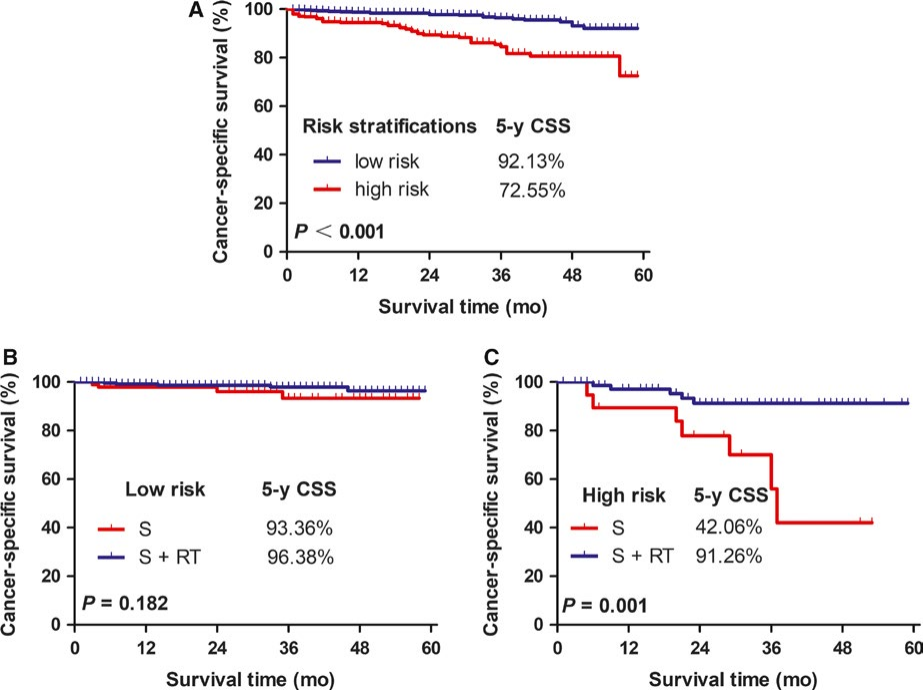
Kaplan‐Meier analysis of cancer‐specific survival according to (A) risk stratifications (low risk vs high risk, *P* < 0.001); (B) radiotherapy (RT) for low‐risk patients (surgery alone (S) vs S  +  RT, *P* = 0.182); and (C) RT for high‐risk patients (S vs S  +  RT, *P* = 0.001)

By weighting each risk variable (to express its relative importance) according to the *β* regression coefficient and Exp(B) derived from the Cox model, we assigned scores to the following prognostic factors: age ≥70 years, 3 points; grade III or IV tumor, 2 points; and <12 nodes resected, 2 points (Table 3). These three scores were added together to obtain a total score for each patient. The Cutoff Finder identified an optimal cutoff value of 2.5 points for our population (Figure 3). According to this cutoff value, 64.3% of the patients were identified as having a low risk of cancer‐related death (total score <2.5), and 35.7% of patients were identified as having a high risk of tumor recurrence (total score ≥2.5). The CSS was significantly better in the low‐risk group than in the high‐risk group (92.13% vs 72.55%, *P* < 0.001; Figure 4A). Furthermore, no survival difference was observed between low‐risk patients who had undergone surgery alone vs those who had undergone surgery plus radiotherapy (93.36% vs 96.38%, *P* = 0.182; Figure 4B). However, among high‐risk patients, the addition of radiotherapy to surgery doubled the CSS (42.06% vs 91.26%, *P* = 0.001; Figure 4C, Table 4).

**Table 3:** Risk variables for the scoring system

Risk variable	Sig.	Exp(B)	Risk coefficient	Risk score
Age	<0.001	3.425		
<70 years			0	0
≥70 years			3.425	3
Tumor differentiation	0.044	2.078		
Grade I/II			0	0
Grade III/IV			2.078	2
Number of nodes resected	0.032	0.544		
<12			0	2
≥12			0.544	0

**Table 4:** Survival analysis of patients stratified to two different risk groups

Risk stratification	Without radiotherapy		With radiotherapy		*P*
	N	5‐year CSS (%)	N	5‐year CSS (%)	
Low‐risk group	101	93.36	264	96.38	0.182
High‐risk group	22	42.06	80	91.26	0.001

CSS, cancer‐specific survival.

## 4 DISCUSSION

To the best of our knowledge, this is the first study to develop a risk‐stratification model with easily available clinicopathological factors to identify a subset of pT3N0 rectal cancer patients who might benefit from adjuvant radiotherapy. Among patients who were classified as having a high risk of cancer‐related death according to this risk‐stratification model, the addition of adjuvant radiotherapy to surgery doubled the CSS (*P* = 0.001), whereas low‐risk patients experienced a negligible survival benefit after undergoing adjuvant radiotherapy (*P* = 0.182).

Previous studies have attempted to explore the role of adjuvant radiotherapy in the management of patients with pT3N0 rectal cancer. Kennecke et al^9^ found that patients treated with perioperative radiotherapy had a superior survival rate over those treated with surgery alone (5‐year disease‐specific survival: 82.7% vs 70.4%, *P* < 0.001). A propensity score‐matched analysis also demonstrated an improved overall survival after additional radiotherapy for patients with stage II or III rectal cancer treated with postoperative chemotherapy (89.9% vs 69.8%, *P* = 0.021).^4^ Similarly, our data showed a superior CSS of 94.40% for the surgery plus radiotherapy group compared to 79.67% for the surgery‐alone group (*P* = 0.001). However, some studies have found no improvement in the local control rate after adjuvant radiotherapy for stage II cancer patients.^12^, ^13^ Another retrospective analysis from our institution has also cast doubt on the benefit of adjuvant radiotherapy for the entire cohort of pT3N0 patients, especially in the era of total mesorectal excision.^14^ Our novel risk‐stratification model successfully identified a subgroup of high‐risk pT3N0 patients who might benefit from adjuvant radiotherapy.

Lymph node evaluation is essential for cancer staging and clinical decisions regarding the administration of adjuvant therapy for colorectal cancer.^15^ A specific “ideal” minimum number of nodes that must be resected for accurate staging and better survival have been debated, with suggested cutoff values of 6‐17 nodes.^16^, ^17^, ^18^ As per the AJCC guidelines, a median of 12 lymph nodes should be examined for colorectal cancer patients undergoing curative surgery.^19^ Lykke et al^20^ retrospectively analyzed the data of 6793 patients from the national database of the Danish Colorectal Cancer Group. The authors reported that among patients with stage I or II cancer who underwent surgery alone, the 5‐year overall survival was 70.4% if fewer than 12 nodes had been resected and 79.2% if 12 or more nodes had been resected (*P* < 0.001).^20^ In the present study, the resection of 12 or more nodes was associated with a survival benefit compared to the resection of fewer than 12 nodes (*P* = 0.018). It has been suggested that as many nodes as possible should be removed in the case of node‐negative disease to avoid understaging.^21^ Vather et al reported that the harvesting of more than 16 nodes was associated with a significant survival benefit for patients with Dukes B colon cancer.^22^ However, no survival advantage was found in our study for >16 nodes examined over 12‐16 nodes examined (*P* = 0.101). Therefore, we suggest that the optimal number of nodes that must be resected is likely to be 12, as this could improve survival without resulting in adverse effects due to extensive lymph node dissection.

Age and tumor differentiation have been reported to be related to oncological outcomes.^9^, ^23^ Consistent with these reports, our study also found that an age ≥70 years and poor tumor differentiation were independent clinicopathological prognostic factors for CSS. A global survival improvement among rectal cancer patients over the last decade has been reported by De Angelis et al^24^; however, no survival improvement has been observed among elderly patients. The worse outcomes in elderly patients might be associated with the lower use of multimodal management for these patients than for their younger counterparts. In our subgroup analysis of adjuvant radiotherapy, 80.13% of patients were younger than 70 years old, while only 19.61% were aged 70 years or more. Tumor differentiation was also defined as a vital factor in our risk‐stratification system. High‐grade colorectal cancers, which have poorly differentiated or undifferentiated cells, present a poorer prognosis than low‐grade or well‐differentiated cancers.^25^

Marital status has been proposed as an important factor associated with oncological outcomes.^26^ Previous reports have suggested that unmarried patients have a worse survival than married patients in the case of various cancers such as head and neck, lung, colorectal, pancreatic, breast, and prostate cancer.^27^ Consistent with this finding, a survival advantage was observed among married patients in our study (*P* < 0.001). Possible reasons for this survival advantage are that married patients are more likely to undergo cancer screening, be diagnosed at earlier stages, experience better financial support, and receive the recommended treatment.^28^, ^29^ In our cohort, radiotherapy was significantly more common among patients who were married than among those who were single or widowed/divorced (*P* = 0.006). Our finding suggests that a patient's marital status should also be noted in clinical practice.

Other studies have sought out additional risk factors for pT3N0 rectal cancer patients. Preoperative carcinoembryonic antigen (CEA) >5 ng/mL and circumferential resection margin (CRM) involvement have been reported to predict adverse oncological outcomes.^30^, ^31^ In our cohort, no statistical difference was observed in patients with elevated vs nonelevated CEA levels, but there was a trend toward a worse CSS in the former group (*P* = 0.055). Furthermore, no survival difference was found between CRM‐positive and CRM‐negative patients (*P* = 0.558).

Some limitations of our study should be noted. First, the SEER database lacked data on local control, which is an important outcome that reflects the effects of radiotherapy. Second, there were no details on whether chemotherapy was administered pre‐ or postoperatively, and this might be a potential confounder in our study. Third, comorbidity data are not recorded in the SEER database; therefore, we selected CSS as an endpoint to decrease any potential bias.

## 5 CONCLUSION

The present study showed that age, tumor differentiation, number of nodes resected, marital status, and radiotherapy were independent prognostic factors for patients with pT3N0 rectal cancer. A novel risk‐stratification system based on these prognostic factors could help to identify patients for whom adjuvant radiotherapy would be beneficial.

## CONFLICT OF INTEREST

The authors report no conflicts of interest in this work.
